# Phytochemical Profile and Selective Cytotoxic Activity of a *Solanum bulbocastanum* Dun. Methanolic Extract on Breast Cancer Cells

**DOI:** 10.3390/plants11233262

**Published:** 2022-11-27

**Authors:** Mihnea Paraschiv, Magda Csiki, Zorita Diaconeasa, Sonia Socaci, Ovidiu Balacescu, Elena Rakosy-Tican, Daniel Cruceriu

**Affiliations:** 1 Department of Molecular Biology and Biotechnology, “Babes-Bolyai” University, 5-7 Clinicilor Street, 400006 Cluj-Napoca, Romania; 2 Faculty of Food Science and Technology, University of Agricultural Sciences and Veterinary Medicine, 3-5 Calea Mănăştur, 400372 Cluj-Napoca, Romania; 3 Department of Genetics, Genomics and Experimental Pathology, The Oncology Institute “Prof. Dr. Ion Chiricuta”, 34-36 Republicii Street, 400015 Cluj-Napoca, Romania

**Keywords:** *Solanaceae*, phenolic acids, alkaloids, volatile compounds, anticancer activity, selective cytotoxicity

## Abstract

*Solanum bulbocastanum* is a wild potato species, intensively used in potato breeding programs due to its resistance to environmental factors. Thus, its biochemical profile and putative human health-related traits might be transferred into potato cultivars aimed for consumption. This study aims to assess the phytochemical profile and the selective cytotoxicity of an *S. bulbocastanum* extract against breast cancer cells. Dry leaves were subjected to ultrasonication-assisted extraction in methanol [70%]. The phenolic and glycoalkaloid profiles were determined by HPLC-PDA/-ESI+-MS. The volatile profile was investigated by nontargeted ITEX/GC-MS. The extract was tested against three breast cancer cell lines (MCF7, MDA-MB-231, HS578T) and a healthy cell line (HUVEC) by the MTT assay, to assess its selective cytotoxicity. The phenolic profile of the extract revealed high levels of phenolic acids (5959.615 µg/mL extract), and the presence of flavanols (818.919 µg/mL extract). The diversity of the volatile compounds was rather low (nine compounds), whereas no glycoalkaloids were identified, only two alkaloid precursors (813.524 µg/mL extract). The extract proved to be cytotoxic towards all breast cancer cell lines (IC_50_ values between 139.1 and 356,1 µg/mL), with selectivity coefficients between 1.96 and 4.96 when compared with its toxicity on HUVECs. Based on these results we conclude that the exerted cytotoxic activity of the extract is due to its high polyphenolic content, whereas the lack of *Solanaceae*-specific glycoalkaloids might be responsible for its high selectivity against breast cancer cells in comparison with other extract obtained from wild *Solanum* species. However, further research is needed in order to assess the cytotoxicity of the individual compounds found in the extract, as well as the anti-tumor potential of the *S. bulbocastanum* tubers.

## 1. Introduction

Plants have been known since prehistory to possess medicinal properties, hence their use in treating a plethora of diseases [1,2], including cancer. Even nowadays, with the advancement of synthetic compound technology, 41.9% of all new drugs approved from 1981 to 2019 were of natural origin or derived from natural products [3], whereas in 2010 alone, 61.7% of all natural medicinal compounds newly discovered were from higher plants [4]. Thus, the plant kingdom represents an important source of such compounds, especially if we consider that only 10% of all plant species were screened for their medicinal activity [5], with potentially many more to possess such properties.

Breast cancer (BC) is the most common type of cancer among women worldwide, with an incidence of over 2 million new cases and a mortality of 0.62 million in 2018, being the fifth cause of cancer-related death overall [6]. BC is considered a highly heterogenous disease, invasive breast cancers being classified into four intrinsic molecular subtypes with distinct clinical outcomes (luminal A, luminal B, Her2 positive and Triple Negative). Luminal BCs are less aggressive and are treated with targeted endocrine therapies along with chemotherapy, having a good prognosis. The 5-year survival rates of Her2-positive BCs have improved substantially over the past decades due to the discovery of anti-Her2-targeted therapies which are nowadays administrated along conventional chemotherapy for this molecular subtype [7]. On the other hand, the triple negative breast cancer subtype (TNBC), representing 10–30% of all BC [8], is the most aggressive subtype, with the worst prognosis, high metastatic potential and no targeted therapies available [9].

Plants are extensively used in breast cancer management. Firstly, plant-derived drugs, especially taxanes, are often part of the conventional chemotherapy regimens for breast cancer patients. Secondly, different compounds and formulations are used as plant-derived supplements in complementary and alternative medicine (CAM), 40–80% of patients with breast cancer reporting the use of some kind of CAM [10,11,12]. Lastly, plants have a role in cancer prevention through nutrigenomics, as diet is proven to be a contributing factor to cancer prevalence [13]. Despite the significant progress made in breast cancer prevention and treatment in the past decades, therapeutic failures are still common in the current clinical practice and the therapeutic options are still limited, especially for TNBC. Therefore, considering the significant unexplored potential of plant-derived compounds in cancer management, intensive research is needed to identify novel phytoconstituents that might be used in cancer treatment and/or prevention.

*Solanum bulbocastanum* is a wild, diploid (2*n* = 24) potato species, native to Mexico. Because of its strong natural resistance to the different potato pathogens, such as aphids [14] or late blight (*Phytophtora infestans*) [15], *S. bulbocastanum* is widely used in potato breeding programs as a genetic source of biotic resistance [14,15]. One of the most important plant resistance mechanisms consists of the synthesis of secondary metabolites, compounds which have a diverse range of biological activities that help the plant survive in harsh conditions, such as pathogenic attacks or environmental stress [16]. One of these biological activities characteristic for some secondary metabolites is toxicity to different kinds of cells, including mammalian cells. In the case of *Solanum* genus, the secondary metabolites known to be cytotoxic are mainly glycoalkaloids and phenolic compounds [17].

Based on the cytotoxicity of alkaloids and phenolic compounds, extracts obtained from different wild *Solanum* species, including *S. nigrum, S. chacoense or S. incanum,* were found to possess anti-tumor activities [18]. However, many such species were not evaluated so far neither for the biochemical profile nor for the antitumor potential. Such an example is *S. bulbocastanum,* no data regarding its cytotoxicity towards cancer cells being available at the moment. Furthermore, in terms of its phytochemical profile, *S. bulbocastanum* was evaluated only for its glycoalkaloid content and the data available in this regard is somewhat contradictory. While some studies report the presence of both α-chaconine and α-solanine in this species at low concentrations [19,20], others were not able to identify these *Solanaceae*-specific alkaloids in *S. bulbocastanum* [21]. Therefore, considering the lack of knowledge regarding this species and its putative antitumor activity based on results obtained with its phylogenetically close relatives, *S. bulbocastanum* might have an impact in cancer management, at least in the prevention of cancer due to its usage in potato breeding programs.

In this context, this study aims to assess the antitumor activity of a *Solanum bulbocastanum* leaf extract against three breast cancer cell lines and one healthy endothelial cell line, in vitro. The BC cell lines belong to both luminal and triple negative breast cancer molecular subtypes in order to take into account the high heterogeneity of breast cancers, whereas the healthy cell line is used to determine the selectivity coefficients of the extract towards cancer cells. Furthermore, the biochemical profile of the extract, in terms of phenolic, alkaloid and volatile compounds is assessed, in a first attempt to identify which constituents are responsible for the observed cytotoxic activity.

## 2. Results

### 2.1. Preparation of S. bulbocastanum Extract

The *S. bulbocastanum* extract was obtained using the ultrasound-assisted extraction (UAE) [22] method and 70% methanol as a solvent. The starting plant material consisted of 0.96 g dried leaves collected from ex vitro cultivated plants and 0.23 g of powder was obtained after the extraction procedure; thus, the UAE extraction method used had a yield of 23.8%. Plants were grown under ex vitro conditions in order to stimulate in planta secondary metabolites synthesis. UAE technique was employed because it was previously proven to be more effective than other classical methods of extraction, in terms of the total amount of extracted compounds [22]. The solvent used was methanol 70%, as it is suitable for extracting a wide variety of polar phytocompounds with high efficiency [23,24].

### 2.2. Phenolic and Alkaloid Profile of the S. bulbocastanum Extract

**Figure 1:**
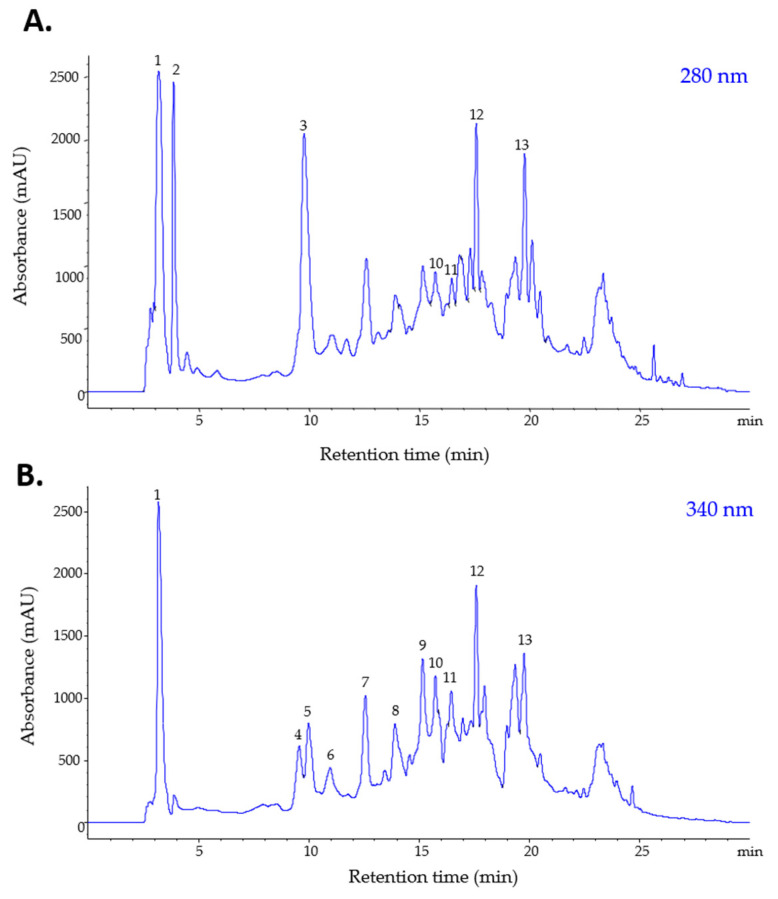
HPLC-PDA chromatograms of phenolics and alkaloids from the *S. bulbocastanum* extract, recorded at 280 (**A**) and 340 nm (**B**).

The tentative identification of individual phenolic compounds and alkaloids was performed by ESI-MS, whereas their quantities were determined by HPLC. In total, 11 phenolic compounds were identified in the methanolic extract of *S. bulbocastanum* leaves, among which 9 were phenolic acids and 2 were flavonols (Figure 1; Table 1). In terms of their quantities, phenolic acids are the major constituents of the extract, with hydroxybenzoic and hydroxycinnamic acids representing 41,8% (2833.8 µg/mL) and 46,1% (3125.8 µg/mL) of the total phenolics found in the extract (Table 1).

Interestingly, no glycoalkaloids specific for the *Solanaceae* family, such as α-chaconine and α-solanine, were identified in the *S. bulbocastanum* methanolic extract obtained from leaves. However, two alkaloid precursors were found in small quantities in the leaves’ extract (Figure 1; Table 1), compounds that were previously reported in other *Solanum* species [25].

### 2.3. Volatile Profile of the S. bulbocastanum Extract

The volatile compounds of the extract were tentatively identified using ITEX/GC-MS, and the relative abundance of each individual compound in the extract was determined using the percentage of each compound’s attributed peak area from the total peak area. In total, nine volatile compounds were found in the methanolic extract obtained from *S. bulbocastanum* leaves, mostly belonging to ketones, esters and aldehydes classes (Table 2). Only one terpenoid was found in the extract, namely eucalyptol, with a rather low relative abundance.

### 2.4. Selective Cytotoxic Activity of the S. bulbocastanum Extract

**Figure 2:**
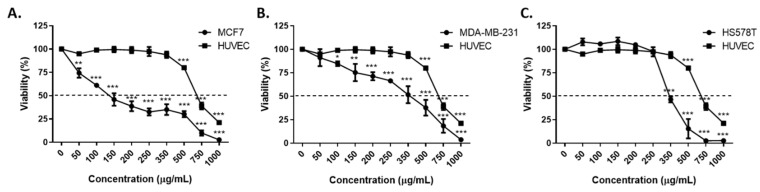
Cytotoxic activity of *S. bulbocastanum* leaves extract against MCF7 (**A**), MDA-MB-231 (**B**) and Hs578T (**C**) in comparison to HUVEC cell lines, at 48 h after administration. The statistical significance for differences between control cells and treated cells at every concentration was assessed by unpaired, two-tailed *t*-test (*p* * > 0.05; *p* ** > 0.01, *p* *** > 0.001).

**Figure 3:**
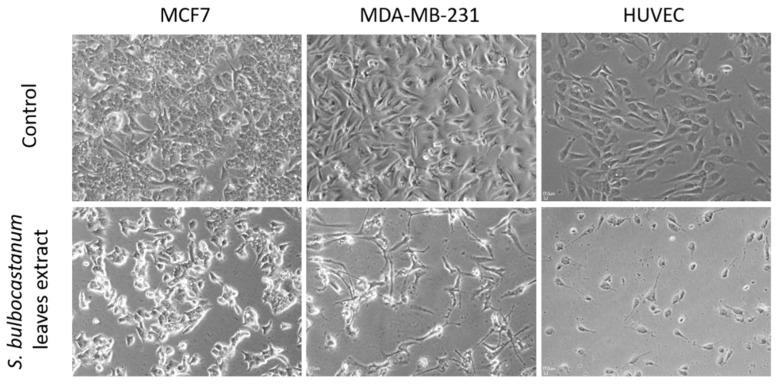
Morphological aspects of MCF7, MDA-MB-231 and HUVEC cells treated with the extract of *S. bulbocastanum* leaves, at a concentration equal to the IC_50_ values, at 48 h after administration.

The extract obtained from the leaves of *S. bulbocastanum* was evaluated for its cytotoxic activity by the MTT assay at 48 h after administration, at nine successive concentrations (50, 100, 150, 200, 250, 350, 500, 750, 1000 µg/mL), against three breast cancer cell lines (MCF7, MDA-MB-231 and Hs578T) a healthy line (HUVEC). The extract was characterized by dose-dependent cytotoxicity on all three breast cancer cell lines (Figure 2). The half maximal inhibitory concentrations (IC_50_ values) varied between 139.1 µg/mL for MCF7 and 351.6 µg/mL for HS578T. (Table 3). Exposure of the cells to the extracts at concentrations equal to the IC_50_ value caused substantial morphological changes in the cells, besides reducing the number of viable cells (Figure 3). By comparing the IC_50_ values obtained for the breast cancer cells and the healthy HUVEC cell line, the extract proved to possess selective cytotoxicity against all three tested cell lines (Table 3). The highest selectivity coefficient in the cytotoxic activity was registered for the luminal breast cancer cell line MCF7, which is five times more sensitive to the extract than HUVECs.

## 3. Discussion

Given the extended usage of *Solanum bulbocastanum* in potato breeding programs due to its various resistances to biotic factors [15], its bioactive constituents may be transferred into potato cultivars and, ultimately, end up in the human diet. Therefore, human health issues, like cancer incidence, may improve, as it is argued that the food we consume is a contributing factor to the initiation and progression of cancer [13]. Therefore, using this perspective as a starting point, this study presents for the first time the phenolic and volatile biochemical profile, and the in vitro antitumor activity of a *S. bulbocastanum* leaf extract. Even though the biochemical profile of the leaves is not identical with the one of tubers aimed for consumption, previous studies performed on other wild *Solanaceae* species show a partial overlap between the two [26] and thus, this study might be a good starting point in the assessment of the overall anti-tumor potential of this species.

The *S. bulbocastanum* methanolic extract obtained from leaves proved to be rich in phenolic compounds, especially in phenolic acids, which represented 87.9% of all polyphenols found in the preparation. Both the phenolic acids, like hydroxycinnamic acids and hydroxybenzoic, and the flavanols identified are reported here for the first time as phytochemical components of *S. bulbocastanum*. Phenolic acids [27], as well as flavonoids, including flavonols [28,29] are described as having strong antitumor activities. Individual phenolic compounds found in the *S. bulbocastanum* extract, such as chlorogenic acid [30,31,32], protocatechuic acid [22,30], quercetin-rutinoside (rutin) [33,34] or quercetin-glucoside [35] are reported to be cytotoxic for various cancerous cells. As such, the phenolic compounds found in the *S. bulbocastanum* extract in high quantities and the interactions between them are most probably responsible for the exerted in vitro antitumor activity of the extract.

The methanolic extract of *S. bulbocastanum* leaves obtained by the UAE technique did not contain any *Solanaceae*-specific glycoalkaloids, such as α-chaconine and α-solanine, which are known to be part of the phytochemical profile of a wide variety of wild *Solanum* species. This data is consistent with previous findings, which demonstrated that *S. bulbocastanum* in particular does not contain such compounds [21] or that if present in its leaves, these metabolites are found in very low quantities [19,20,36]. In any case, the presence of such glycoalkaloids in the final extract also depends on the extraction method and solvent used, and thus these results do not exclude the possibility that these *Solanaceae*-specific glycoalkaloids are found in the leaves of *S. bulbocastanum.* On the other hand, two unnamed alkaloid precursors were identified in the extract. These compounds were previously reported in other *Solanum* species: one with a mass of 456.347 and the H_46_C_29_O_3_N formula and one with the mass of 460.378 and the H_50_C_29_O_3_N formula [25]. Considering that no glycoalkaloids were identified in the methanolic extract and the low concentrations for the alkaloid precursors, we hypothesize that this category of compounds does not participate in the overall toxicity of the extract on human cells.

The volatile biochemical profile of *S. bulbocastanum* is reported for the first time in this study. However, the diversity of the volatile compounds found in the methanolic extract of this species is rather low, with eucalyptol being the only terpenoid identified. Eucalyptol is also the only constituent found in the extract that was previously described to have cytotoxic activity [37,38]. Thus, we can conclude that volatile compounds play a minor role, if any, in the in vitro antitumor activity of the extract.

The extract of *S. bulbocastanum* leaves proved to be significantly cytotoxic towards breast cancer cells, with the lowest IC_50_ value for MCF7 cell line. This paper is the first study that demonstrates the in vitro antitumor activity of *S. bulbocastanum* preparations. The better results against MCF7 cell line in terms of the response to the extract administration were expected, this cell line being a less aggressive one, whereas MDA-MB-231 and HS578T are triple negative cell lines, characterized by intermediate to low chemotherapy responsiveness [39]. However, the extract was selective in the cytotoxic activity towards all three BC cell lines when compared with its toxicity against a healthy human endothelial cell line. Therefore, these results highlight a first premise for the potential of *S. bulbocastanum* formulations in cancer prevention and/or therapy.

Previously, a similar experimental design in terms of extraction procedure, characterization of the biochemical profile of the extract and analysis of the in vitro cytotoxic activity was carried out for a phylogenetically related species of *S. bulbocastanum*, the wild species *S. chacoense* [26]. Interestingly, the extracts obtained from the leaves of both species are cytotoxic to the BC cell lines to approximately the same degree according to the IC_50_ values, but the toxicity exerted on the healthy human endothelial cell line is more than two times higher for the *S. chacoense* extract (*S. chacoense*-IC_50_ = 328.8 µg/mL; *S. bulbocastanum-*IC_50_ = 689.9 µg/mL). Therefore, *S. bulbocastanum* extract is much more selective in the cytotoxic activity towards cancer cells than the extract obtained from the leaves of *S. chacoense*, and thus this emphasizes once again its potential in cancer prevention and/or treatment. In an attempt to understand this significant difference between the cytotoxicity of the two extracts, a comparison between the biochemical profile of the two species is needed. The *S. bulbocastanum* extract is richer in phenolic compounds, both in terms of their diversity and quantities (8–9 times higher concentration for the total polyphenolic content), but it has no glycoalkaloids, which are present in high concentrations in *S. chacoense* [26]. Taking into consideration the protective effects of phenolic compounds [40], and the known toxicity of potato glycoalkaloids [41] on human healthy cells, we hypothesize that these differences in the biochemical profile of the two species might be a starting point for explaining the differences observed regarding their toxic effects on healthy human endothelial cells.

Nonetheless, further research is needed in order to exhaustively assess the human health-related potential *Solanum bulbocastanum* possesses, with regard to cancer implications and nutrigenomics. Firstly, the biochemical profile and antitumor potential of the *S. bulbocastanum* tubers needs to be assessed too. Secondly, phytoconstituents found in the *S. bulbocastanum* extract should be individually tested for their cytotoxic activity on cancerous cells lines to identify which are responsible for the extract’s cytotoxic activity and its selectivity against cancer cells. The synergistic, additive, and antagonistic interactions between these compounds should also be evaluated. Thirdly, the potato hybrids (*S. tuberosum* × *S. bulbocastanum*) obtained in potato breeding programs need be biochemically characterized to evaluate to what extend the phytochemical profile of the wild species is transferred into the cultivated species, especially with regard to tubers.

## 4. Materials and Methods

### 4.1. Plant Material and Culture Conditions

*Solanum bulbocastanum* Dunal in vitro grown plants, accession GLKS-31741 (blb41), were provided by Gross Lüsewitz Potato Collections (GLKS) of the IPK Gene Bank, Leibniz— Institute of Plant Genetics and Crop Plant Research, Germany. The plants were cultivated in vitro [temperature: 21 °C; photoperiod: 16 h/8 h (light/dark); PPF-photosynthetic photon flux levels: 135 μmol m^−2^ s^−1^; light spectrum: 380–680 nm], in the growing room, on RMB5 medium [standard MS medium [42] enriched with Gamborg B5 vitamins [43], 0.7% agar, pH = 5.8]. After four weeks of in vitro culture, plants were transferred ex vitro, in soil, for eight weeks, under the same controlled laboratory conditions as the in vitro plants, with watering three times a week. Leaves were collected, dried until they were of a crunchy texture, and then powdered for extract preparation.

### 4.2. Cell Line and Culture Conditions

Three breast cancer (BC) cell lines (MCF7-luminal BC; MDA-MB-231 and HS578T-triple negative BC) and one healthy cell line (HUVEC-human umbilical vein endothelial cells) were used in this study. MCF7 cells were cultured in MEM media, MDA-MB-231cells in RPMI-1640 media, Hs578T cells in DMEM media and HUVECs in ECM media. All media were supplemented with 10% fetal bovine serum (FBS), 1% glutamine and 1% penicillin-streptomycin. Additionally, the Hs578T medium was supplemented with 0.01 mg/mL insulin, whereas 1% non-essential amino-acids (NEAA) were added in the MCF7 media. Cells were maintained in a humidified atmosphere containing 5% CO_2_, at 37 °C, in a cell culture incubator. All cell lines were obtained from the European Collection of Authenticated Cell Cultures (ECACC).

### 4.3. Preparation of Plant Extract

The protocol used for obtaining the plant extract, based on an ultrasound-assisted extraction (UAE) method, was adapted from Cruceriu et al. [26]. Briefly, over the powdered dried plant material (0.96 g), 70% methanol was added, at a liquid-to-solid ratio of 10:1 mL/g. The resulting solution was thoroughly vortexed, sonicated at an ultrasonic power of 750 W, at 40 °C, in 3 cycles of 10 min each (Sonics Vibra-cell VC750; Sonics & Materials Inc., Newtown, CT, USA) and further macerated for 24 h, in the dark. After maceration, the homogenate was centrifuged at 450× *g* for 5 min, and the supernatant was collected. Solvent was added again on the remaining pellet and centrifugation was repeated in order to ensure that all the polar phytochemicals were extracted. The reunited supernatants were filtered through a 0.45 µm Millipore filter (Burlington, MA, USA) to make sure that all the contaminants were removed. Subsequently, the raw methanolic extract was concentrated to powder using a vacuum rotary evaporator at 40 °C, under reduced pressure (Heidolph Laborota 4000 Efficient; Heidolph Instruments GmbH & CO, Schwabach, Germany). The dry residues were resuspended with either 70% ethanol for biochemical analysis, or in dimethyl sulfoxide (DMSO) for the in vitro experiments, both at a concentration of 100 mg/mL (stock solution). Both stock extracts were filtered through a 0.22 μm Millipore, before testing.

### 4.4. HPLC-PDA/-ESI-MS Identification and Quantification of Phenolic Compounds and Alkaloids

The quantification of phenolic compounds and alkaloids was performed by HPLC analysis using an Agilent 1200 system (Chelmsford) with quaternary pump delivery system LC-20 AT (Prominence), a degasser DGU-20 A3 (Prominence) and a diode array SPD-M20 UV–VIS detector (DAD), whereas identification of the compounds was performed by mass spectrometry with a mass detector single-quadrupole Agilent model 6110 (Agilent Technologies, Santa Clara, CA, USA) equipped with an ESI probe.

Compound separation was performed using an Eclipse XDB C18 column (4.6 × 150 mm, 5 μm), Agilent Technologies for 30 min, at 25 °C, with a 0.5 mL/min flow rate and an injection volume of 20 μL. Before injecting, all the samples were filtered through a PTFE filter (13 mm i.d., 0.22 μm). The mobile phases consisted of solvent A-distilled water and 0.1% acetic acid and solvent B-acetonitrile and 0.1% acetic acid. The gradient elution system was designed in the following manner: 0–2 min, isocratic with 5% (vol/vol) eluent B; 2–18 min, linear gradient from 5% to 40% (vol/vol) eluent B; 18–20 min, linear gradient from 40% to 90% (vol/vol) eluent B; 20–24 min, isocratic with 90% (vol/vol) eluent B; 24–25 min, linear gradient from 90% to 5% (vol/vol) eluent B; 25–30 min, isocratic with 5% (vol/vol) eluent B. The chromatograms were monitored at 280 nm corresponding to hydroxybenzoic acids and 340 nm for hydroxycinnamic acids, flavonoids and alkaloids. The quantification of compounds was conducted based on their retention times, UV-VIS spectra, and comparison with commercial standards (chlorogenic acid, caffeic acid, ferulic acid, α-solanine, α-chaconine) all purchased from Sigma-Aldrich (St. Louis, MO, US), and published data. Mass spectrometry analysis was designed in the following way: measurements were performed in the positive mode with a capillary voltage of 3000 V, a temperature of 300 °C and a collision energy of 50 eV. Data were collected in full scan mode within the range of 100 to 1000 m/z, with a 8 L/min nitrogen flow-rate. The data acquisition and interpretation were performed using Agilent ChemStation software. For the quantification of the compounds, 3 standard curves were used: gallic acid (concentrations between 5 and 100 µg/mL) for hydroxybenzoic acids, chlorogenic acid (concentrations between 10 and 50 µg/mL) for hydroxycinnamic acids and alkaloids and rutin (concentrations between 10 and 100 µg/mL) for flavonols.

### 4.5. Non-Targeted ITEX/GC-MS Headspace Analysis of Volatile Compounds

Volatile compounds were identified in the headspace of the methanolic extract by in-tube extraction technique (ITEX) coupled with gas chromatography mass spectrometry. The extract was put in a headspace vial and incubated at 60 °C for 20 min. Afterwards, the volatile compounds were extracted from the headspace phase using a Combi PAL AOC-5000 autosampler (CTC Analytics) with a headspace syringe ITEX-II equipped with a microtrap (ITEX-2TrapTXTA, Tenax TA 80/100 mesh) and directly thermally desorbed into the chromatograph injector. The analysis was carried out on a GCMS QP-2010 (Shimadzu Scientific Instruments) model gas chromatography mass spectrometer. The volatile compounds were separated on a Zebron ZB-5 ms capillary column (30 m × 0.25 mm) and 0.25 μm film thickness. The column temperature was set at 30 °C (hold for 2 min), increased to 140 °C with a rate of 5 °C/min and then increased to 270 °C and hold for 5 min. The carrier gas used was helium, with a flow rate of 1 mL/min and a split ratio of 1:20. The injector, ion-source and interface temperatures were set at 250 °C. The MS detection used for the qualitative analysis was performed on a quadrupole mass spectrometer operating in the electron ionization mode (EI) at 70 eV. The volatile compounds were recorded in full scan mode (40–450 m/z), in total ion chromatogram (TIC). The volatile compounds were identified by first comparing the mass spectrometric information of each chromatographic peak to NIST27 and NIST147 mass spectra libraries (considering a minimum similarity of 85%), and by comparison with retention indices drawn from www.pherobase.com or www.flavornet.org. The relative percentage of each compound was estimated as a fraction of its integrated ion area from total ion chromatograms (TIC) area (100%).

### 4.6. Selective Cytotoxicity Assay

The cytotoxic activity of the *S. bulbocastanum* extract was evaluated on all four cell lines by the MTT assay (Sigma-Aldrich), according to the manufacturer protocol. Briefly, cells were seeded in 96-well plates at a density of 2 × 10^4^ cells/well and left to adhere for 24 h. The extract was added in nine successive concentrations, varying from 50–1000 µg/mL, in six technical replicates and left on cells for 48 h. After supernatant removal, 100 µL of the MTT solution (1 mg/mL) was added onto the cells and left for incubation for 1 h. After the MTT solution was removed, 150 µL DMSO was used to solubilize the formed formazan and the absorbance was measured at a wavelength of 570 nm, using a microplate spectrophotometer (Synergy HTX, BioTek, Winooski, VT, USA). All experiments were performed in triplicate.

Based on the absorbance values, viability was calculated as the fraction of viable cells compared with the untreated control. The half maximal inhibitory concentration (IC_50_) values on each cell line were calculated in GraphPad Prism Software Version 7 (GraphPad Software, Inc. Avenida de la Playa, La Jolla, San Diego, CA, USA). The selectivity coefficient in the anti-tumor action was calculated as the ratio between the IC_50_ value on the healthy cell line (HUVEC) and the IC_50_ value obtained for each cancer cell line.

## 5. Conclusions

The methanolic extract obtained by the UAE technique from *S. bulbocastanum* leaves was rich in phenolic compounds, especially phenolic acids, whereas it was characterized by a rather low diversity of volatile constituents. Interestingly, it did not contain any *Solanaceae*-specific glycoalkaloids, such as α-chaconine and α-solanine, even though 2 alkaloid precursors were identified in the preparation at low concentrations. This is the first time a comprehensive analysis of the biochemical profile of *S. bulbocastanum* was performed.

The extract of *S. bulbocastanum* proved to be cytotoxic towards breast cancer cells in vitro, with stronger effects registered for luminal breast cancer. Furthermore, it was selective in the in vitro antitumor activity against both luminal and triple negative breast cancer cells, when compared with its toxicity on healthy human endothelial cells. This is the first time an extract of *S. bulbocastanum* was tested for its cytotoxic activity in vitro, on cancer cells. By comparing the extract’s cytotoxic effects with those of other *Solanum* related species, we hypothesize that the high selectivity against cancer cells is due to the high polyphenolic content along with the lack of glycoalkaloids in the preparation. However, further studies are necessary in order to identify which compounds are truly responsible for the selective cytotoxicity reported here.

## Figures and Tables

**Table 1:** Tentative identification and quantification of phenols and alkaloids in the methanolic extract of *S. bulbocastanum* leaves. R_t_—retention time; [M + H]^+^—molecular ion; UV λmax—wavelengths of maximum absorption in the visible region.

Class of Compounds	Peak No.	R_t_ (min)	[M + H]^+^ (m/z)	UV λ_max_ (nm)	Compound	Concentration (µg/mL Extract)
Hydroxybenzoic acid (BA)	1	3.21	139	265	Hydroxybenzoic acid	1238.708
	2	3.86	156	260	Dihydroxybenzoic acid	596.786
	4	9.77	156	260	Protocatechuic acid	998.314
**Total BA ^1^**						2833.808
Hydroxycinnamic acid (HBA)	3	9.56	369	330	3-Feruloyquinic acid	337.601
	5	9.98	355	322	3-Caffeoylquinic acid (Neochlorogenic acid)	644.398
	6	10.96	355	322	4-Caffeoylquinic acid (Cryptochlorogenic acid)	305.146
	7	12.55	355	322	5-Caffeoylquinic acid (Chlorogenic acid)	671.983
	8	13.89	181	322	Caffeic acid	476.366
	9	15.15	369	330	5-Feruloyquinic acid	690.313
**Total HBA ^2^**						3125.807
Flavonols (FL)	10	15.75	611	360, 255	Quercetin-rutinoside (Rutin)	452.452
	11	16.29	465	360, 255	Quercetin-glucoside	366.467
**Total FL ^3^**						818.919
Alkaloid (ALK)	12	17.61	467	370,320,230	Alkaloid precursor	441.431
	13	19.76	450	420,310,240	Alkaloid precursor	372.093
**Total ALK ^2^**						813.524

^1^ expressed as µg gallic acid/mL; ^2^ expressed as µg chlorogenic acid/mL; ^3^ expressed as µg rutin/mL.

**Table 2:** Tentative identification and relative quantification of volatile compounds in the methanolic extract of *S. bulbocastanum* leaves. R_t_—retention time; Relative abundance—relative percentage (%) of total peaks area. SI—similarity in comparison with NIST27 and NIST147 mass spectra libraries.

Peak No.	Compound	R_t_ (min)	Relative Abundance (%)	Area	Height	SI
1	Acetic acid, butyl ester	6.081	85.23	42,403,774	7,960,501	99
2	Benzaldehyde	11.723	0.26	130,981	82,780	78
3	Phenol	12.368	0.97	484,938	130,120	99
4	Eucalyptol	14.249	0.43	212,757	77,371	95
5	Acetophenone	15.464	1.98	987,044	296,437	99
6	Butanoic acid, 2-methyl-, 3-methylbutyl ester	16.654	0.26	129,757	56,950	95
7	Butanoic acid, 3-methyl-, 3-methylbutyl ester	16.874	1.48	738,417	288,419	100
8	n-Amyl isovalerate	16.941	0.62	309,632	135,954	99
9	Ethanone, 1-[4-(1-methylethyl)phenyl]-	22.789	8.76	4,356,596	1,174,963	100

**Table 3:** The IC_50_ values and the selectivity coefficients in the antitumor action for *S. bulbocastanum* leaves extract against MCF7, MDA-MB-231, Hs578T and HUVEC cell lines, at 48 h after administration. The IC_50_ values are means for three biological replicates.

Cell Line	*S. bulbocastanum* Leaves Extract	
	IC_50_ (µg/mL)	Selectivity Coefficient
MCF7	139.1	4.96
MDA-MB-231	336.4	2.05
Hs578T	351.6	1.96
HUVEC	689.9	-

## Data Availability

Not applicable.
